# Magnetic Resonance Enterography Findings of Intestinal Behçet Disease in a Child

**DOI:** 10.1155/2017/8061648

**Published:** 2017-05-24

**Authors:** Tommaso D'Angelo, Romina Gallizzi, Claudio Romano, Giuseppe Cicero, Silvio Mazziotti

**Affiliations:** ^1^Section of Radiological Sciences, Department of Biomedical Sciences and Morphological and Functional Imaging, University of Messina, Policlinico “G. Martino”, Via Consolare Valeria 1, 98100 Messina, Italy; ^2^Department of Pediatrics, University of Messina, Policlinico “G. Martino”, Via Consolare Valeria 1, 98100 Messina, Italy

## Abstract

Behçet's disease (BD) is a multisystem disorder of unknown aetiology, characterized by recurrent oral ulcers, genital ulcers, uveitis, skin lesions, and pathergy. Gastrointestinal disease outside the oral cavity is well recognized and usually takes the form of small intestinal ulcers, with the most significant lesions frequently occurring in the ileocaecal region. Symptoms usually include nausea, vomiting, colicky abdominal pain, and change in bowel habit and it is not unusual that patients may present late, with life-threatening complications requiring surgery. Diagnosis has been hindered for many years by limitations in imaging the small bowel and it is usually achieved by means of endoscopy and CT of the abdomen. Magnetic resonance enterography (MRE) is a relatively new technique, which has a high diagnostic rate in patients with Crohn's disease (CD). Although many similarities between CD and intestinal BD have already been described in literature, the role of MRE in the evaluation of intestinal BD has never been defined up to now. We report a case of a 12-year-old female patient with diagnosis of BD who presented at our institution for recurrent colicky abdominal pain and diarrhoea. The patient underwent MRE that demonstrated the gastrointestinal involvement.

## 1. Introduction

Gastrointestinal Behçet's disease (BD) outside the oral cavity is well recognized and may lead to life-threatening complications, such as bowel perforation or fistulization, which may require bowel resection surgery. It usually takes the form of deep, circular ulcers that can potentially occur in all the gastrointestinal tract, with the most common involvement of the ileocaecal region.

Patients usually complain of colicky abdominal pain, nausea, and change in bowel habit. Differential diagnosis with Crohn's disease (CD) may be difficult, with several extraintestinal manifestations, such as oral ulcers and arthralgia, which can be recognized in both diseases.

Imaging of intestinal BD has been hindered for many years by the inherent limitations of the radiological techniques used to study the small bowel. In fact, only wireless capsule endoscopy and CT have been able to adequately image the gastrointestinal involvement of BD up to now [1, 2]. MR-Enterography (MRE) is a relatively new imaging technique, which has already demonstrated a good diagnostic value for small bowel pathology, particularly in CD. However, no data has ever been reported regarding the usefulness of MRE in patients with intestinal BD [1].

We describe a case in which a 12-year-old female patient with intestinal BD underwent MR-Enterography that allowed a better evaluation of the small bowel involvement.

## 2. Case Presentation

A 12-year-old female patient, with history of recurrent oral aphthosis, was originally admitted at our university hospital in September 2014 for progressively increasing pancranial headache, stiff neck, left oculomotor paresis, left facial nerve paresis, unstable gait, and left hemiparesis. The laboratory findings showed neutrophilic leukocytosis, increased C-reactive protein (CRP), and raised erythrocyte sedimentation rate (ESR). She presented papilledema and retinal vasculitis, which led to loss of visual acuity in both eyes. The lumbar puncture revealed high pressure of the cerebrospinal fluid (CSF) and oligoclonal bands of IgG, which were found also in the blood serum. MRI of the brain confirmed the papilledema, showing a bilateral budging of the optic disc into the vitreous chamber. It showed prominent subarachnoid space around the optic nerves and a partial empty sella turcica, compatible with diagnosis of pseudotumour cerebri.

The patient also presented gastrointestinal symptoms such as colicky abdominal pain, nausea, and diarrhoea and she reported an episode of gastrointestinal bleeding. A colonoscopy was performed, revealing round-shaped ulcers along the caecal region. In addition, esophagogastroduodenoscopy showed several aphthous ulcerations along the gastric body.

Diagnosis of BD with neurological and gastrointestinal manifestations was made and the patient was treated with intravenous bolus injection of methylprednisolone for the first three days, followed by oral administration of prednisone and cyclophosphamide. Three months later, the latter was replaced with mycophenolate mofetil.

After relief of neurological and gastrointestinal symptoms, steroids dose was progressively reduced with a concomitant relapsing of gastrointestinal symptoms, such as abdominal pain and fever.

In December 2015, the same patient presented to our institution to undergo MRE, which was suggested to evaluate the small bowel.

To perform the magnetic resonance of the small bowel, the patient was asked to fast from solids and liquids for 4–6 hours prior to the study and to assume a 1200 mL of polyethylene glycol (PEG) solution within 45 minutes before the beginning of the scan. MRE was performed using a 1.5 T MR imaging system (Gyroscan Achieva, Philips, Best, Netherlands) and phased-array abdominal coils. The standard protocol that we applied included various T2-weighted pulse sequences along axial and coronal planes. True fast imaging with steady-state (True-Fisp; TR/TE: 4.20/2.10 ms, FA: 60°) and half-Fourier acquisition single-shot turbo spin echo (HASTE; TR/TE: ∞/80 ms) with and without fat-suppression were also performed as well as diffusion-weighted sequences, using a diffusion factor b fixed at 0, 400, and 800 s/mm^2^.

**Figure 1:**
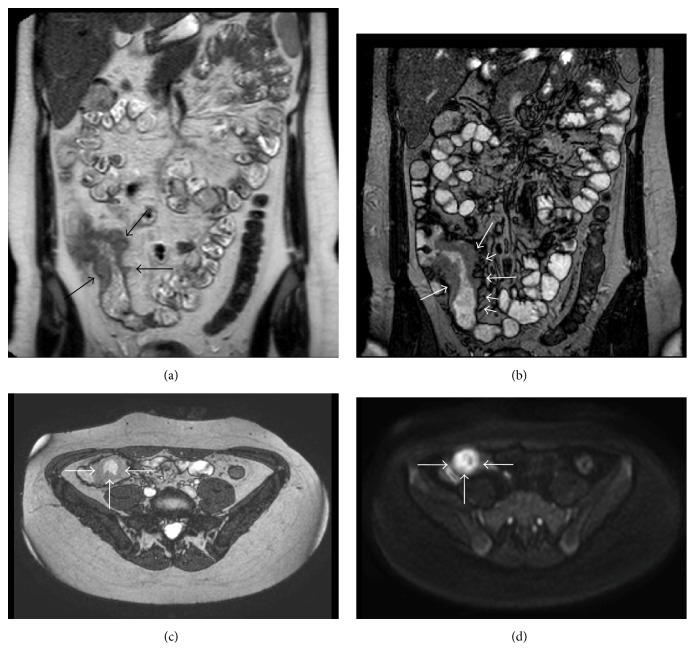
Diffuse bowel wall thickening with polypoid appearance* (long arrows)* and ectatic vasa recta* (short arrows)* are well demonstrable on coronal HASTE (a) and True-FISP (b) MR images as well as on axial True-FISP MR image (c). Axial diffusion-weighted MR image, performed at *b*-value of 800 s/mm^2^, shows restricted diffusion of water within the affected bowel wall (d).

MRE showed marked inflammatory changes of the terminal ileum and caecum, characterized by diffuse bowel wall thickening with polypoid appearance. Diffusion-weighted images demonstrated restricted diffusion of water within the affected bowel wall. Mesenteric vascular engorgement was also seen (Figure 1).

**Figure 2:**
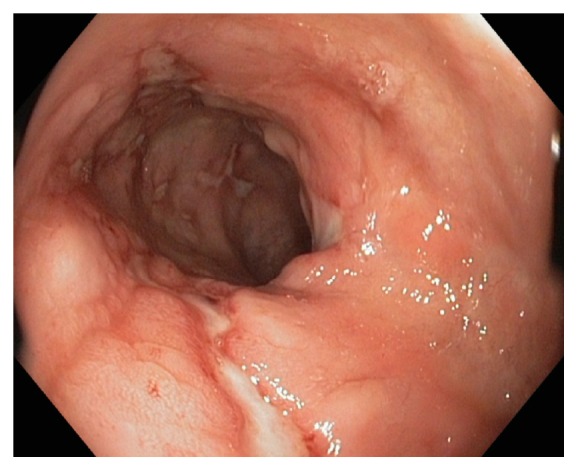
Endoscopic image of the caecal region shows deep, large, and oval ulcerations.

These findings were also confirmed by the endoscopic evaluation of the ileocaecal region that showed oedema, erythema, and round-shaped ulcers (Figure 2). The patient started therapy with adalimumab, with rapid relief of symptoms.

In March 2016, gastrointestinal symptoms relapsed again and the patient was planned for elective terminal ileum resection surgery. However, a few days prior to the expected day of surgery, the same patient came to emergency room with intense and acute abdominal pain. Emergency CT scan showed bowel perforation in adjacencies of the diseased terminal ileum and the girl rapidly underwent bowel resection surgery.

**Figure 3:**
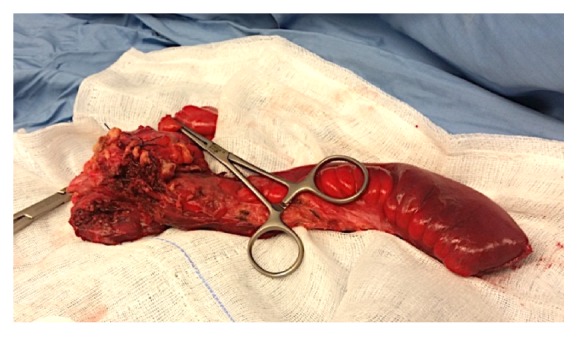
Surgical specimen reveals multiple punched out ulcerations along a diffusely thickened terminal ileum.

The surgical specimen revealed multiple punched out ulcerations along a diffusely thickened terminal ileum (Figure 3). Histological examination of the surgically resected intestinal specimens showed thickening of the blood vessel wall and infiltration of inflammatory cells in the vascular wall and perivascular area (i.e., neutrophils and mononuclear cells), findings consistent with submucosal phlebitis.

## 3. Discussion

BD is classified among inflammatory vascular diseases, affecting arterial and venous vessels of all kinds and sizes. The etiopathogenesis of the disease remains still unknown, but the combination of genetic factors affecting the immune regulation and undetermined environmental triggers may explain the variability of disease expression. The disease distribution is worldwide, even if there is a higher prevalence in populations living along the ancient “Silk-Route,” which extends from eastern Asia to the Mediterranean basin [3]. Although the disease rates and the clinical expression vary to some extent by ethnic origin, recurrent mucocutaneous lesions, skin lesions, ocular findings, and reactivity of the skin to needle prick or injection (pathergy test) constitute the most common clinical hallmarks of BD. In addition, a wide spectrum of findings can be observed, with frequent involvement of neurologic, genitourinary, mucocutaneous, and gastrointestinal systems. Diagnosis is made when typical symptoms occur, and it is primarily based on sets of clinical criteria. Recently, the International Team for the Revision of the International Criteria for Behçet's disease (ICBD) has suggested a new criteria/point score, which markedly improved sensitivity and diagnostic accuracy over the older International Study Group (ISG) criteria still maintaining a comparable specificity [3, 4]. Our clinical case encompassed one major and one minor ISG criteria for diagnosis of BD and had a score of five according to ICBD point-score system, allowing diagnosis of BD (Table 1).

In addition, several studies have demonstrated that gastrointestinal symptoms (i.e., chronic diarrhoea and proctorrhagia) are significantly correlated with BD and when associated with typical intestinal lesions (e.g., round-shaped ulcers, deep ulcers with discrete margins, and inflammatory pseudopolyps) they configure a clinical condition also known as intestinal BD. Intestinal BD is more frequent in East Asia (i.e., Japan, Taiwan, and Korea) than in other areas of the world [3]. Factors associated with poor patient's prognosis are younger age, high ESR and CRP, and low albumin concentration at diagnosis [5].

Clinical course of intestinal BD is usually mild and patients uncommonly experience a disease flare-up. However, some patients may have a severe clinical course, undergoing frequent disease flare-ups and requiring corticosteroid therapy, immunosuppressant therapy, or surgical treatment [5].

The most typical form of gastrointestinal manifestation of BD is the ulceration of the ileocecal region, which may result in a high frequency of complications, such as fistulization, perforation, bleeding, and peritonitis. Up to now, diagnosis and monitoring of patients with intestinal BD have been done with endoscopy, while radiological examinations have had a limited role [2].

In fact, although conventional enteroclysis is able to detect mucosal abnormalities of the small bowel, this fluoroscopic technique is not widely used since it is relatively invasive and often unpleasant for the patient. Since the last decade, Computed Tomography Enterography (CTE) has been widely used in patients with gastrointestinal manifestations of BD, showing a significant diagnostic value and depicting most of intra- and extraluminal abnormalities [6].

At CTE, the typical findings of gastrointestinal BD are a thickening of bowel wall, consisting in mural edema with ulcer penetration, or a “tumor-like” polypoid mass, both usually showing a marked mural hyperenhancement due to the vascular pathologic substrate [7].

The chronic relapsing nature of intestinal BD requires patients to undergo repeated diagnostic imaging sessions due to high frequency of complications. Several studies have recently demonstrated that patients with intestinal BD are exposed to high levels of radiation owing to an excessive use of abdominal CT scans [1].

MRE is a relatively new imaging technique, which has demonstrated a good diagnostic value for small bowel pathology [8–10]. In particular, it is currently considered part of the standard evaluation algorithm in the follow-up of CD patients, thanks to its capacity to finely depict pathological alterations of the small bowel and to detect structured segments. In addition, MRE is a radiation-free, noninvasive technique, well-tolerated also by younger patients.

In our case, MRE showed a diffuse bowel wall thickening of the terminal ileum and caecum with polypoid appearance and restricted water diffusivity on DWI, a characteristic feature of active inflammation.

Due to similar CTE and MRE findings, the main differential diagnosis includes CD.

Although a multitude of authors have already assumed that intestinal BD may mimic CD clinically, pathologically, endoscopically, and even radiologically, the role of MRE in detecting intestinal BD has never been reported up to now [1, 3, 11].

MRE may have a primary role in patients with history of BD for staging, follow-up, and also depicting extraluminal complications.

In conclusion, gastroenterologists and radiologists should be aware of the similar CTE and MRE findings of CD and intestinal BD. Although CTE has some advantages compared to MRE, such as a better spatial resolution and a lower acquisition time, if validated by larger studies MRE may represent a useful diagnostic tool to evaluate patients with gastrointestinal manifestations of BD, sparing them an excessive and inappropriate radiation exposure.

## Figures and Tables

**Table 1:** International criteria for Behçet's disease, point-score system: scoring ≥ 4 indicates Behçet's diagnosis [4].

Sign/symptom	Points
Ocular lesions	2
Genital aphthosis	2
Oral aphthosis	2
Skin lesions	1
Neurological manifestations	1
Vascular manifestations	1
Positive pathergy test^*∗*^	1^*∗*^

^*∗*^Pathergy test is optional and the primary scoring system does not include pathergy testing. However, where pathergy testing is conducted one extra point may be assigned for a positive result.
